# Breaking point: Case series of tendon ruptures in Hemodialysis patients

**DOI:** 10.12688/f1000research.155799.2

**Published:** 2024-12-12

**Authors:** Muhammed Ehsan Nazeer, Dr Askhar Haphiz, Dr Muhammed Nazeer, Dr Pradeep Moni, Dr Praveen Muraleedharan

**Affiliations:** 1 Trauma and Orthopaedics, North Cumbria integrated care trust,, Cumberland Infirmary, Carlisle, England, UK; 2 Trauma and arthroscopy, MS Orthopedicsorthopaedics, P.B.No.1, Anayara P.O, Trivandrum, KIMSHEALTH, Thiruvananthapuram, Kerala, 695029, India; 3 Consultant, Trauma and Group coordinator orthopaedics, MS Orthopedics, P.B.No.1, Anayara P.O, FRCS (Glasgow) KIMSHEALTH, Trivandrum, Kerala, 695029,, India; 4 Resident, Trauma and orthopaedics, MBBS, P.B.No.1, Anayara P.O,, KIMSHEALTH, Thiruvananthapuram, Kerala, 695029, India; 5 Nephrology, Senior consultant, DM Nephrology P.B.No.1, Anayara P.O, – Kerala, India, KIMSHEALTH, Trivandrum, Kerala, 695029, India

**Keywords:** ESRD, Quadriceps, Hyperparathyroidism, Tendon rupture, Hemodialysis, Case series

## Abstract

**Introduction:**

Spontaneous tendon ruptures in end stage kidney disease patients have the potential to cause long- term morbidity, and timely intervention is required to prevent complications that can severely affect the functional status of the patient.

**Case presentation:**

A series of six tendons (two triceps tendons and two bilateral patellar tendons) in three patients with ESKD undergoing hemodialysis is discussed in this case series. Patients were aged 61, 44 and 26 years, and on hemodialysis for 5, 10 and 5 years, respectively.

**Conclusion:**

End -stage kidney disease is associated with a multitude of physiological changes, and the musculoskeletal system is no exception to this. Spontaneous tendon rupture is a multifactorial complication of ESKD, with serious implications for mobility and quality of life. As a result, these patients require a multifaceted approach to ensure optimum results and an early return to activity. We report a series of 6 spontaneous tendon ruptures in 3 patients with ESKD at our institution. We would like to outline the methods of repair for each case and further attempt to assess biochemical parameters that may have contributed to the disease process.

## Introduction

Spontaneous tendon rupture is rare in the general population. Most cases of tendon rupture occur secondary to trauma or degenerative changes resulting from obesity or old age. Other etiologies of tendon rupture include pathological alterations in the substance of the tendon itself, such as gout, ^1^ autoimmune arthritis, ^1^ and end stage renal disease. ^2^ Long-term use of drugs such as statins, quinolones and steroids ^3^ constitute yet another often implicated cause of spontaneous tendon ruptures.

Literature on spontaneous tendon ruptures in patients with ESKD undergoing hemodialysis is uncommon and studies on the exact underlying mechanism and management guidelines for such patients are sparse.

The objective of this case series was to report a series of patients on hemodialysis for ESKD who experienced spontaneous tendon ruptures with emphasis on the methods and results of surgical management. An assessment of laboratory parameters was also performed to identify risk factors for the same.

## Case reports

### Case 1

**Figure 1.**
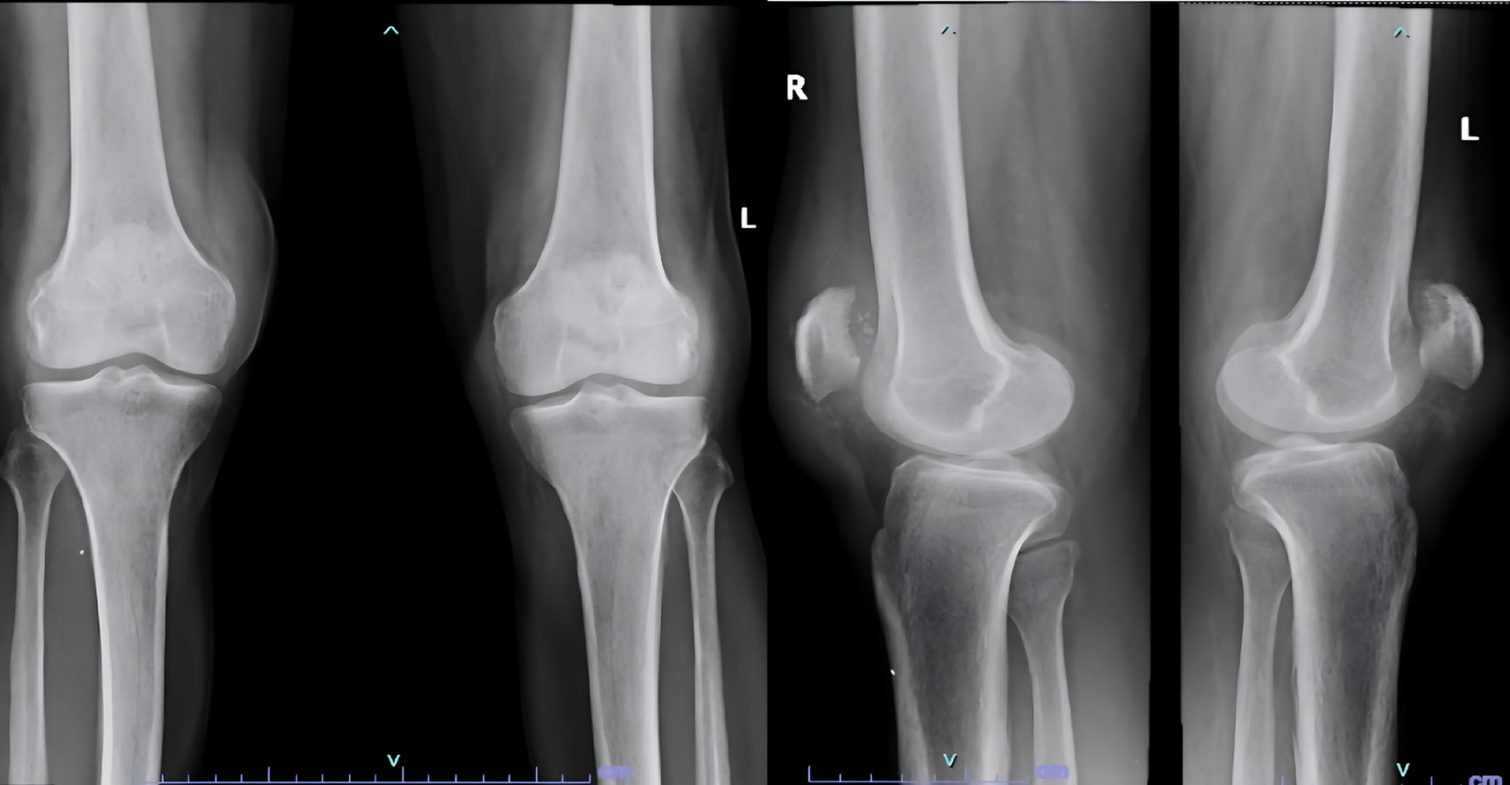
Patella alta noted with calcified patellar tendon.

A 61 year old male suffering with ESKD due to nephrosclerosis on hemodialysis for the last 5 years presented to the emergency department with complaints of acute onset pain and swelling in both knees while attempting to stand up from a sitting position. Examination revealed effusion in both knees with a palpable defect in the bilateral patellar tendons and bilateral extension lag. Lateral radiographs showed bilateral patella alta with calcified patellar tendons ( Figure 1).

**Figure 2.**
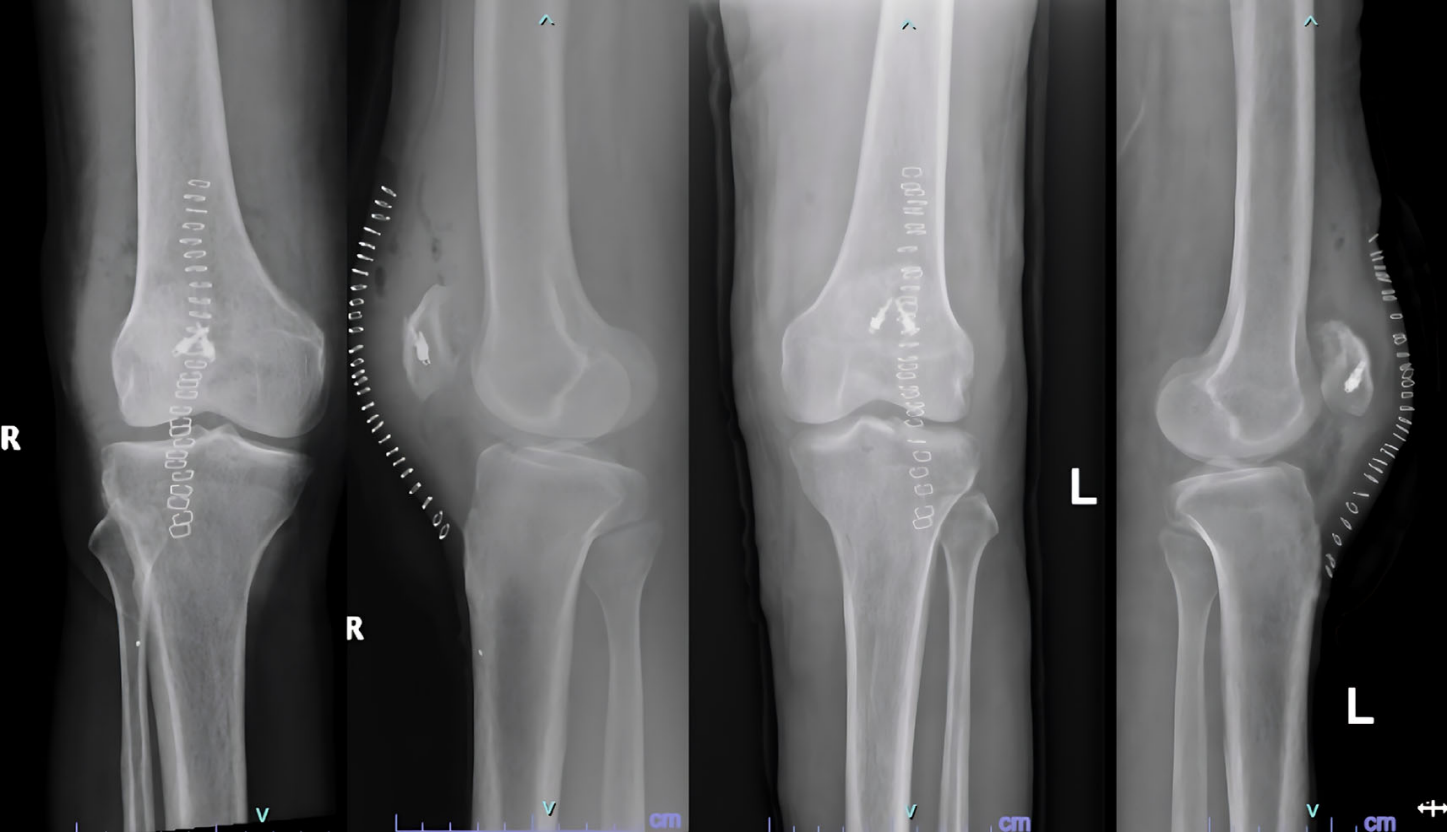
Post operative radiographs after fixation of tendons with suture anchors.

Surgical repair was performed in a staged fashion with an interval of 2.5 weeks in view of his physiological status. Surgical exploration revealed friable tendon edges with partial avulsion and mid substance tear patterns. The degenerated tissue was excised, and the ends of the tendon were reattached to the patella using suture anchors and non-absorbable sutures, followed by closure of the defects in the medial and lateral paratenon using bioabsorbable sutures ( Figure 2).

Post operatively the knees were immobilized in full extension for 6 weeks. DVT prophylaxis was administered using low molecular weight heparin (40 mg, sub-cutaneous injections, once daily) during the hospital stay and Rivaroxaban on Discharge. He was started on active Knee ROM and quadriceps strengthening exercises at six weeks.

He complained of left elbow extension weakness in his review for suture removal on the second knee, and further evaluation revealed a spontaneous rupture of the triceps tendon. This was likely caused by sustained forces while using a walker to assist ambulation. He has been advised to undergo surgical repair, for which he is yet to be reported.

Follow-up examinations at 8 and 8.5-months post-op respectively, examination revealed a knee ROM of 0-120 degrees with bilateral full extension and no residual weakness in the quadriceps mechanism.

End stage renal disease was managed by our nephrology department with thrice weekly hemodialysis sessions and regular monitoring of renal parameters. The patient has been receiving hemodialysis for the past five years.

### Case 2

**Figure 3.**
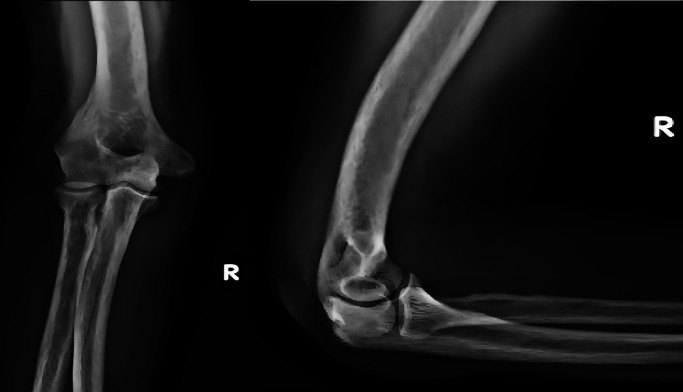
Anteroposterior and lateral radiographs of Case 2 with avulsed triceps tendon.

A 44-yr old male with ESRD secondary to immunoglobinA nephropathy with a history of allograft rejection during three weekly hemodialysis sessions for 10 years presented with complaints of right elbow extension weakness following trivial trauma. Physical examination revealed swelling of the right elbow with inability to activate the elbow extensor mechanism. Radiographs showed an avulsed triceps tendon ( Figure 3).

**Figure 4.**
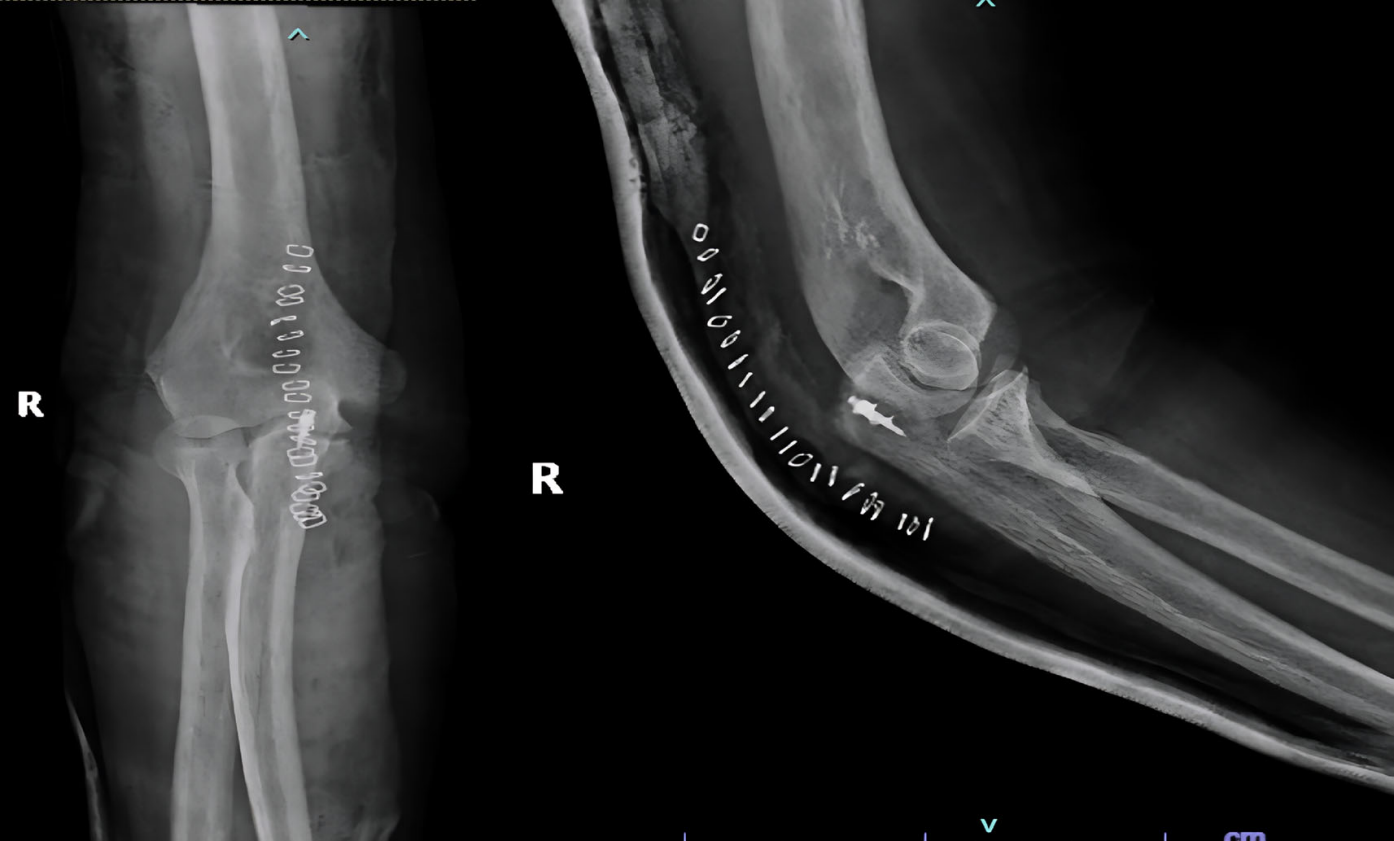
Post-operative radiographs after fixation of tensons using suture anchors.

He underwent open repair of the triceps tendon with a suture anchor inserted in the olecranon after excision of the devitalized tongue of the tissue ( Figure 4). The elbow was immobilized in an above elbow slab at approximately 110° for 6 weeks, after which active mobilization was started. The last follow-up at 7 months revealed grade 5/5 power with full elbow ROM.

### Case 3

A 26-year-old male, suffering from lupus nephritis presented to ER with acute loss of extension in both knees and inability to ambulate following a fall while descending stairs at his house. The treatment history was significant for the intake of methylprednisolone for the last 3 months and twice weekly hemodialysis for ESKD for a duration of 5 years.

**Figure 5.**
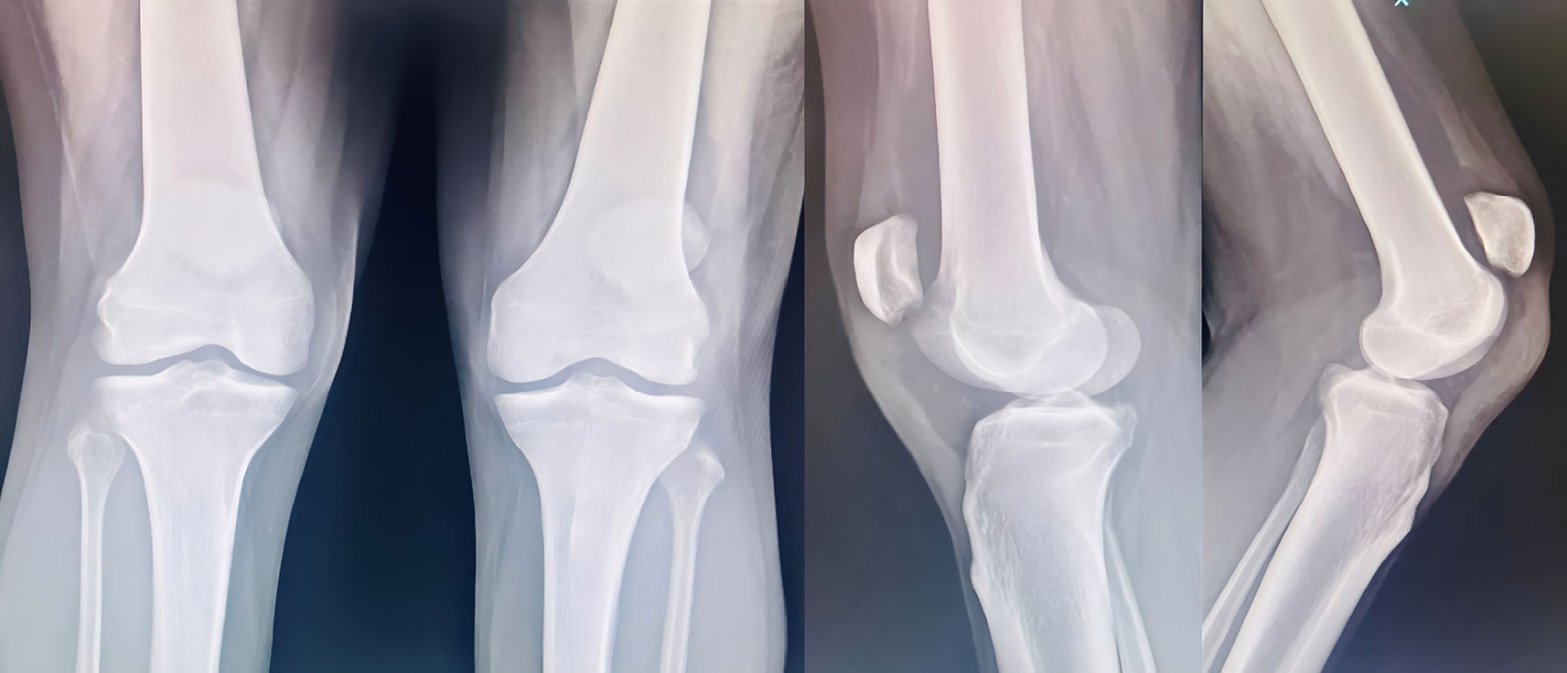
Patella Alta noted in case 3 due to patellar rupture.

Examination revealed bilateral patella alta and boggy swelling in both knees with grade 0 power in bilateral knee extension. Imaging revealed isolated patella alta without any avulsed bony edges ( Figure 5).

**Figure 6.**
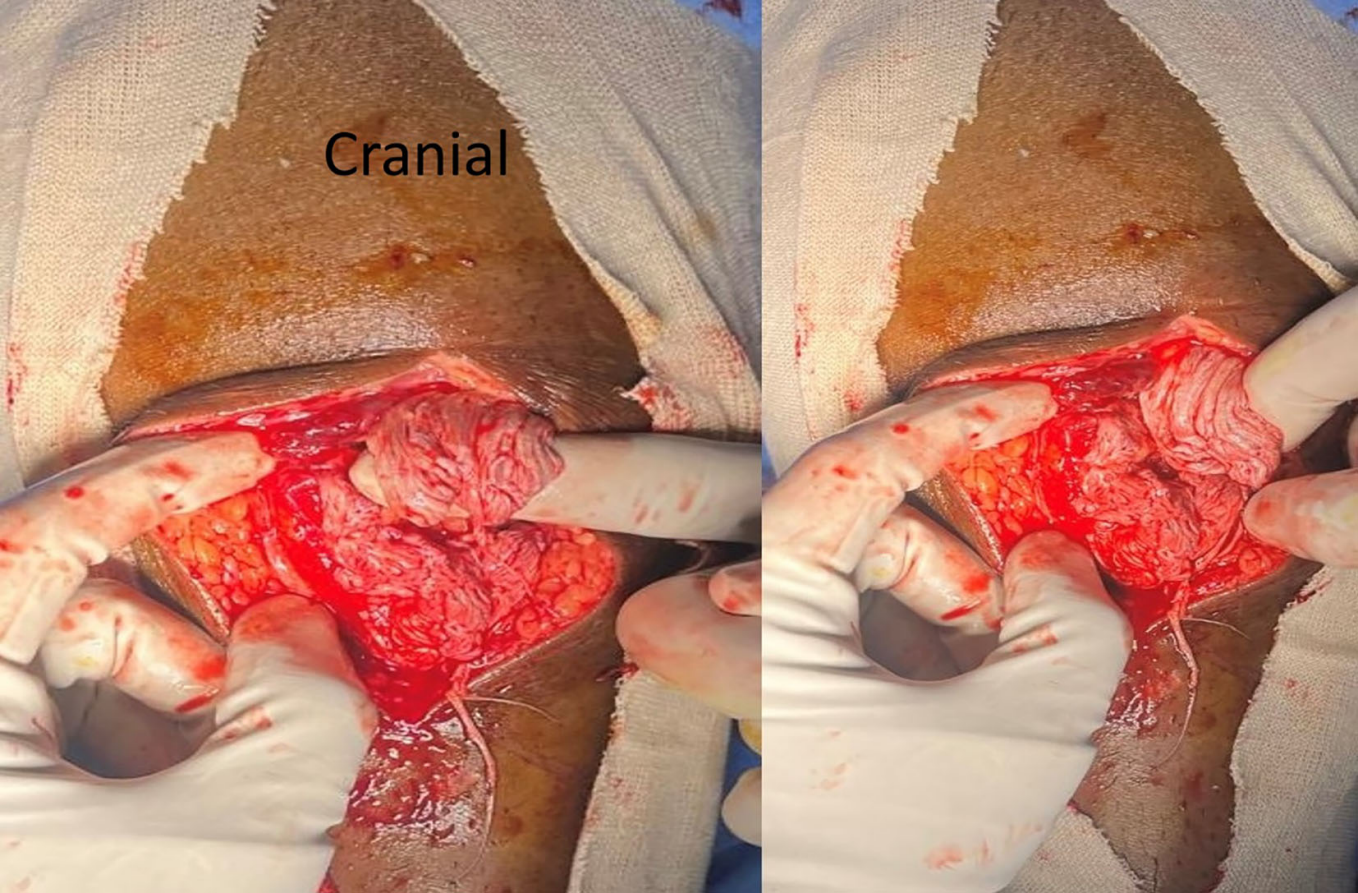
Frayed ends of patellar tendon noted in case 3.

**Figure 7.**
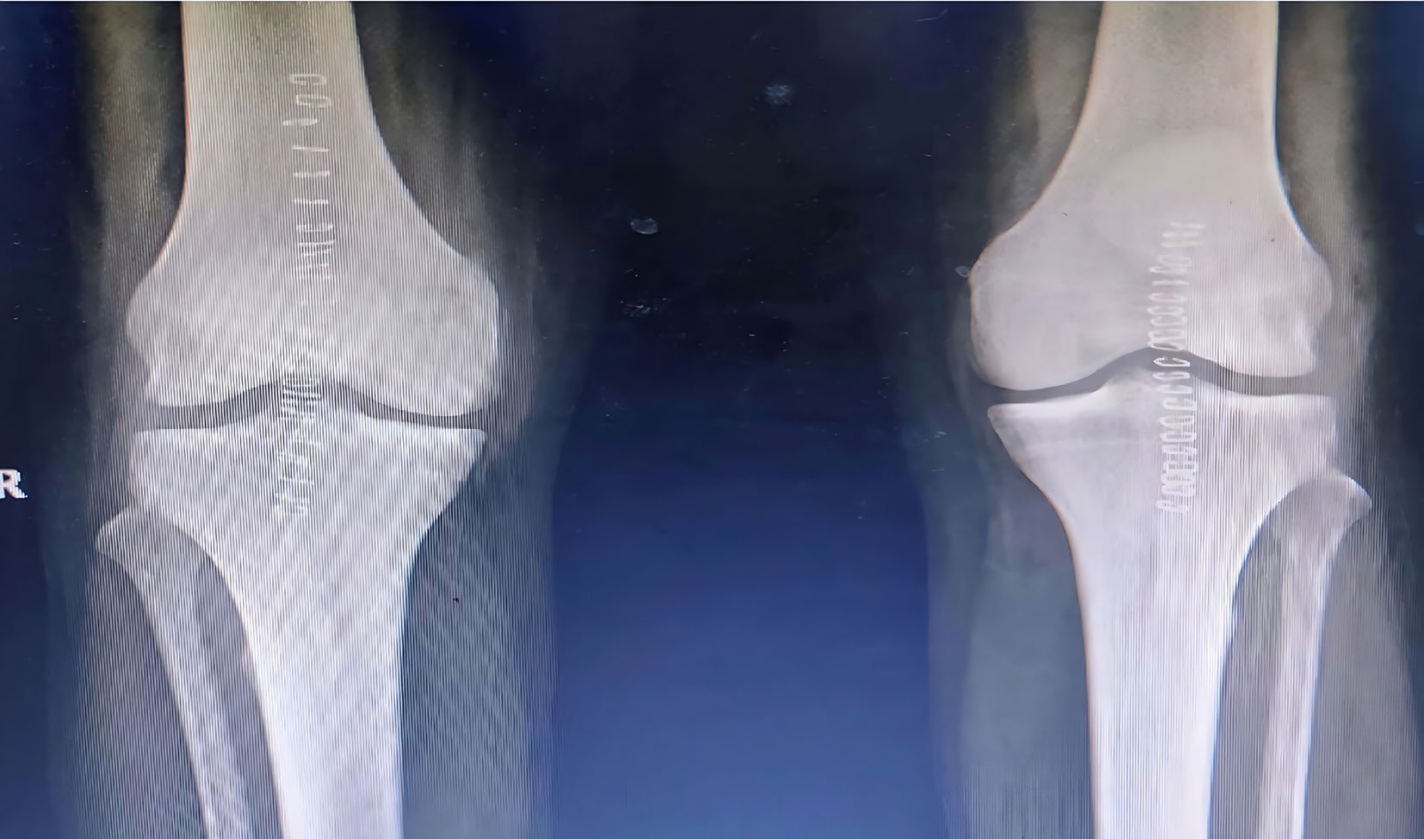
Post operative radiographs of 3 after patellar tendon repair.

He was diagnosed with bilateral patellar tendon rupture and underwent open repair during which severe fraying of the tendons was noted ( Figure 6). Robust repair was performed using non-absorbable sutures in a mattress pattern ( Figure 7). He was given bilateral long knee immobilizers for 6 weeks to protect the repair, following which he was started on assisted ambulation with walker support over the next 4 weeks. At the final review at 6 months, he had grade 5/5 power in both his knee extensors with no appreciable lag on either side.

## Discussion

Normal tendons are strong, with Ultimate Tensile Strengths ranging from 45 to 125 MPa which exceed three times the strain caused by muscle contraction. ^4^ The tendency for spontaneous tendon ruptures in patients with ESKD has often been attributed to five main factors:

1) elastosis secondary to chronic acidosis, ^5^
2) chronically elevated blood urea levels, ^1^
3) amyloidosis with beta 2 microglobulin deposition, ^6^
4) increased cortical bone resorption because of secondary hyperparathyroidism, ^2^
5) high circulating calcium levels resulting in dystrophic calcification of the tendon. ^7^

This contrasts with other cases of spontaneous tendon rupture, which is a condition of middle-aged overweight patients, with bilaterality being an exception rather than the norm. ^8^

A quick diagnosis is paramount because early surgical intervention is a key factor in ensuring optimal results. ^9^ This rare diagnosis should always be considered when dealing with sudden unexplained motor deficits in the lower limbs. Hemarthrosis can further complicate the matter by making it difficult to identify infrapatellar defects. Such cases warrant the use of incongruous techniques, such as ultrasound and MRI to confirm the diagnosis.

A prolonged duration of hemodialysis appears to be a reliable risk factor for the development of spontaneous tendon ruptures. ^10^ The markedly elevated PTH and ALP levels in our patients were also concurrent with the existing literature and explain the avulsed tendon ends noted both intraoperatively and radiographically in 2 cases in our series ( Table 1).

**Table 1.** Lab results of cases showing elevated PTH and ALP levels.

Parameter	Case 1	Case 2	Case 3	Normal range
Hemoglobin	7.6	11.2	8.4	13-16 g/dL
Calcium	8.2	8.3	8.1	8.6-10.2 mg/dL
PTH	1101	1826	1220	15-65 pg/mL
Phosphorous	5.5	4.5	4.9	2.7-4.5 mg/dL
Albumin	3.2	4.4	3.3	3.5-5.2 g/dL
ALP	200	1399	866	40-129 U/L

The repairs were performed as soon as fitness could be obtained, in view of the compromised physiological status of the patients. No additional procedures were required to compensate for retraction or shortening of tendons. Repair was performed using suture anchors and heavy non absorbable sutures. The patients attained full active extension in all cases at an average of 2 months from the date of surgery with no re-ruptures or residual weakness at a minimum 6 months follow up.

Although the exact mechanism of injury remains unknown, most existing studies implicate sub-tendon bone resorption due to secondary hyperparathyroidism, ^2^ in addition to chronic inflammation, as evidenced by reduced serum hemoglobin and albumin levels, which was noted in 50% of cases.

## Conclusion

Spontaneous tendon rupture in patients with ESKD remains a significant complication with considerable potential to cause disability. Robust repair with adequate immobilization and protection during the healing phase is instrumental in ensuring reliable results. Moreover, these patients require cautious physio rehabilitation, including careful walker- assisted mobilization to prevent further tendon ruptures during the postoperative period.

Optimization of renal parameters, such as hyperparathyroidism and hypoalbuminemia, may help improve outcomes and prevent recurrence. Overall, a high index of suspicion for spontaneous tendon rupture is advised in patients with ESKD presenting with acute-onset limb weakness to prevent long-term morbidity.

## Consent

The patients have given their informed consent for the case series to be published. Written consent was obtained for the same.

## Data Availability

All data underlying the results are available as part of the article and no additional source data are required.
