# Supplementary material for: Breaking point: Case series of tendon ruptures in Hemodialysis patients
Source: F1000Res. 2024 Dec 12;13:1121. Originally published 2024 Oct 3. [Version 2] doi: 10.12688/f1000research.155799.2 (PMC11809646; doi:10.12688/f1000research.155799.2)
Supplement: Peer review history

# Reviewer report: Reviewer response for version 2

**DOI:** 10.5256/f1000research.174955.r353382

**Published:** 2024-12-28

**Authors:** Nijil Vasukutty

**Affiliations:** 1 United Lincolnshire Hospitals NHS Trust, Lincoln, England, UK

In this paper, the authors report on a series of six cases with spontaneous tendon ruptures due to chronic renal disease

 The have reported on  a variety of cases with the involvement of quadriceps and triceps.

 This is not an easily recognised condition and a high index of suspicion will be required to diagnose this in a timely fashion. 

 Any delay in diagnosis will make the surgical procedure technically challenging and the outcomes suboptimal.

 The authors do give a brief description of their diagnostic methods, surgical procedures and post-operative rehabilitation with intermediate term outcomes.

 There is a  section  on the biochemical mechanism behind tendon rupture  in end stage renal disease

 This paper will form a valuable addition to the literature on this subject as they look at a different ethology to this difficult problem. 

 This is a short series but that would be expected as this is not a common problem.

 The first line of introduction may be revised as  most degenerate ruptures do occur spontaneously like in the Achilles and these are not rare .

 Avoid using abbreviations the first time this term is referred to as in ESKD 

 Case reports 

 Could the authors clarify whether they used any three dimensional imaging like MRI or ultrasound before undertaking surgery or did they just depend on plain x-rays? 

 In case three the authors are report that the end of the patella tendon  were frayed . In such a situation were the frayed ends excised and if so, how did they make up the lost length as the patella tendon may not be amenable to stretching.

 In the discussion session it may be helpful to add a short paragraph on other causes of spontaneous tendon rupture like use of  Ciprofloxacin and  degenerate tendon rupture.

Are enough details provided of any physical examination and diagnostic tests, treatment given and outcomes?

Yes

Is the background of the cases’ history and progression described in sufficient detail?

Yes

Is sufficient discussion included of the importance of the findings and their relevance to future understanding of disease processes, diagnosis or treatment?

Yes

Is the conclusion balanced and justified on the basis of the findings?

Yes

Reviewer Expertise:

Trauma and Orthopaedics

I confirm that I have read this submission and believe that I have an appropriate level of expertise to confirm that it is of an acceptable scientific standard.

# Reviewer report: Reviewer response for version 2

**DOI:** 10.5256/f1000research.174955.r348090

**Published:** 2024-12-16

**Authors:** Sandeep Vijayan

**Affiliations:** 1 Additional Professor, Department of Orthopaedics, Kasturba Medical College Manipal,, Manipal Academy of Higher Education, Manipal, India

No further comments to make.

 Add (ESKD) after it is mentioned first time in the introduction.

 Correct spelling of tendon in figure 4 legend

Are enough details provided of any physical examination and diagnostic tests, treatment given and outcomes?

Partly

Is the background of the cases’ history and progression described in sufficient detail?

Yes

Is sufficient discussion included of the importance of the findings and their relevance to future understanding of disease processes, diagnosis or treatment?

Yes

Is the conclusion balanced and justified on the basis of the findings?

Yes

Reviewer Expertise:

NA

I confirm that I have read this submission and believe that I have an appropriate level of expertise to confirm that it is of an acceptable scientific standard.

# Reviewer report: Reviewer response for version 2

**DOI:** 10.5256/f1000research.174955.r348092

**Published:** 2024-12-13

**Authors:** Matthew Abramowitz

**Affiliations:** 1 Albert Einstein College of Medicine, Bronx, New York, USA

Lack of description of the dialysis prescriptions remains a limitation.

Are enough details provided of any physical examination and diagnostic tests, treatment given and outcomes?

No

Is the background of the cases’ history and progression described in sufficient detail?

No

Is sufficient discussion included of the importance of the findings and their relevance to future understanding of disease processes, diagnosis or treatment?

Partly

Is the conclusion balanced and justified on the basis of the findings?

Yes

Reviewer Expertise:

physical function, muscle physiology in CKD

I confirm that I have read this submission and believe that I have an appropriate level of expertise to confirm that it is of an acceptable scientific standard.

# Reviewer report: Reviewer response for version 1

**DOI:** 10.5256/f1000research.171011.r335309

**Published:** 2024-11-12

**Authors:** Matthew Abramowitz

**Affiliations:** 1 Albert Einstein College of Medicine, Bronx, New York, USA

This case series brings attention to the risk of tendon rupture in patients with ESKD. There are several areas in which it could be strengthened:

 1.    To improve the generalizability of this report to other patients with ESKD, it would be helpful to present details of the patients’ dialysis prescriptions, such as use of high flux dialyzers (which would reduce the likelihood of beta-2 microglobulin as an etiologic factor), pre-dialysis bicarbonate levels, Kt/V, dialysate settings (especially bicarbonate), and treatment of hyperparathyroidism. If beta-2 microglobulin levels were measured, those data should be presented.

 2.    Regarding case 3, the duration of dialysis should be presented. If the patient was not recently started on dialysis and did not have significant residual kidney function, it would be helpful to know why he was only receiving dialysis twice per week.

 3.    The abstract describes quadriceps tendon ruptures but the case descriptions report patella tendon ruptures. This should be clarified.

 4.    Abstract, Case presentation: the years on hemodialysis should be presented in the same order as the patients’ ages.

 5.    It would be helpful to further discuss potential mechanisms by which risk factors in ESKD may impact tendon physiology and structure.

Are enough details provided of any physical examination and diagnostic tests, treatment given and outcomes?

No

Is the background of the cases’ history and progression described in sufficient detail?

No

Is sufficient discussion included of the importance of the findings and their relevance to future understanding of disease processes, diagnosis or treatment?

Partly

Is the conclusion balanced and justified on the basis of the findings?

Yes

Reviewer Expertise:

physical function, muscle physiology in CKD

I confirm that I have read this submission and believe that I have an appropriate level of expertise to confirm that it is of an acceptable scientific standard, however I have significant reservations, as outlined above.

# Reviewer report: Reviewer response for version 1

**DOI:** 10.5256/f1000research.171011.r335311

**Published:** 2024-11-07

**Authors:** Luis Marcelo Malta

**Affiliations:** 1 Universidade Federal Fluminense, Rio de Janeiro, Brazil

The authors show their experience with a series of tendon ruptures on regular-based hemodialysis patients, addressing aspects of surgical treatment and functional results.

 Despite the fact that it is a case series, giving it a less favorable view in terms of scientific strength or evidence, the study brings important information about the understanding and management of this unusual occurrence.

 There are also some details I would like to point:

   1- Abstract says that the authors report on two bilateral QUADRICEPS ruptures, but both case 1 and 3 are PATELLAR tendon ruptures. Maybe it was just a typing mistake but should be corrected to prevent confusion.

 2- All cases include physical evaluation, both pre and postoperative, without clarifying which scale or functional score was used to do such measurements. I consider this is a minor but important point to address.

 3- Discussion is well done but could include more recent articles at surgical repair aspects and risk factors associated with ruptures available in the literature. I consider this a major point to be revised, because there are much work documented on the field and it would make this section more complete.

Are enough details provided of any physical examination and diagnostic tests, treatment given and outcomes?

Partly

Is the background of the cases’ history and progression described in sufficient detail?

Yes

Is sufficient discussion included of the importance of the findings and their relevance to future understanding of disease processes, diagnosis or treatment?

Partly

Is the conclusion balanced and justified on the basis of the findings?

Yes

Reviewer Expertise:

Orthopaedics / orthopedic surgery

I confirm that I have read this submission and believe that I have an appropriate level of expertise to confirm that it is of an acceptable scientific standard, however I have significant reservations, as outlined above.

# Reviewer report: Reviewer response for version 1

**DOI:** 10.5256/f1000research.171011.r332004

**Published:** 2024-11-06

**Authors:** Sandeep Vijayan

**Affiliations:** 1 Additional Professor, Department of Orthopaedics, Kasturba Medical College Manipal,, Manipal Academy of Higher Education, Manipal, India

**Abstract – **case presentation: better to mention as 'patellar tendon' as the involved part is infrapatellar and not suprapatellar.

**Page 3- case 2: **I am not sure if it is correct to say grade 0 power. Here we are dealing with mechanical disruption of the muscle without loss in power of the muscle. Attempt to move the elbow will definitely contract the triceps muscle without the force transmitting across the tear. So, is it truly grade 0 power in the muscle ?

**Page 3, case 2: **was there any USG or MRI images available confirming the diagnosis and assess the quality of the tissue.

 Was histopathological examination of the excised tissue performed to understand the type of degenerative changes commonly happening in tendons and ligaments in ESRD.

**Page 5, figure 5 legend - **better to mention as 'patellar tendon rupture'.

**Page 6, figure 6-** Please add cranial end and caudal end in the picture for easy understanding for the readers, The picture on the left side appear skewed (stretched horizontally). Please re-size it correctly.

**Page 7- discussion** - It is interesting to note that in most of the reported literatures. it the quadriceps / patellar tendon which is getting ruptured. In your review of literature, did you come across any specific reasoning from the affection of quadriceps tendon in particular.

 Few more minor corrections are suggested. Same has been commented on in the main manuscript and may be accessed by opening the manuscript with Adobe reader.

Are enough details provided of any physical examination and diagnostic tests, treatment given and outcomes?

Partly

Is the background of the cases’ history and progression described in sufficient detail?

Yes

Is sufficient discussion included of the importance of the findings and their relevance to future understanding of disease processes, diagnosis or treatment?

Yes

Is the conclusion balanced and justified on the basis of the findings?

Yes

Reviewer Expertise:

NA

I confirm that I have read this submission and believe that I have an appropriate level of expertise to confirm that it is of an acceptable scientific standard, however I have significant reservations, as outlined above.
